# Revisiting perioperative chemotherapy: the critical importance of targeting residual cancer prior to wound healing

**DOI:** 10.1186/1471-2407-9-118

**Published:** 2009-04-22

**Authors:** William W Harless

**Affiliations:** 1 Tunnell Cancer Center, Lewes, DE 19971, USA

## Abstract

**Background:**

Scientists and physicians have long noted similarities between the general behavior of a cancerous tumor and the physiological process of wound healing. But it may be during metastasis that the parallels between cancer and wound healing are most pronounced. And more particularly and for the reasons detailed in this paper, any cancer remaining after the removal of a solid tumor, whether found in micrometastatic deposits in the stroma or within the circulation, may be heavily dependent on wound healing pathways for its further survival and proliferation.

**Discussion:**

If cancer cells can hijack the wound healing process to facilitate their metastatic spread and survival, then the period immediately after surgery may be a particularly vulnerable period of time for the host, as wound healing pathways are activated and amplified after the primary tumor is removed. Given that we often wait 30 days or more after surgical removal of the primary tumor before initiating adjuvant chemotherapy to allow time for the wound to heal, this paper challenges the wisdom of that clinical paradigm, providing a theoretical rationale for administering therapy during the perioperative period.

**Summary:**

Waiting for wound healing to occur before initiating adjuvant therapies may be seriously compromising their effectiveness, and patients subsequently rendered incurable as a result of this wait. Clinical trials to establish the safety and effectiveness of administering adjuvant therapies perioperatively are needed. These therapies should target not only the residual cancer cells, but also the wound healing pathway utilized by these cells to proliferate and metastasize.

## Background

Cancer has been described as a "wound that won't heal' [1], and general similarities between the behavior of a cancerous tumor and wound healing have been recognized for over a century [2]. Normal wound healing is an adaptive process that requires the integration of multiple distinct cellular behaviors, including cell-cell dissociation; cellular proliferation and migration; angiogenesis; matrix degradation and synthesis; and cell survival during the anchorage independent conditions associated with cellular migration [3]. Diverse and specialized cells – including epithelial cells, fibroblasts, endothelial cells, inflammatory cells, and bone marrow derived progenitor cells – orchestrate this complex process in a temporally coordinated pattern to repair damaged tissue [3]. They communicate with each other through a myriad of cytokines, chemokines and growth factors that act in both a paracrine and autocrine manner and that are predictably released after tissue damage and during the three distinct phases of the wound healing process [4-6].

The first phase of wound healing occurs immediately after tissue injury and lasts for approximately 3 days. This is the period of hemostasis and inflammation. Tissue injury leads to platelet activation and the triggering of the coagulation cascade, resulting in the release/formation of various inflammatory mediators such as platelet activating factor, thrombin, and histamine[6]. Inflammatory mediators trigger arteriolar vasodilation and an increase in microvascular permeability and they activate the expression of endothelial and inflammatory cell surface adhesive molecules such as the selectins and the integrins, which enhance the ability of circulating inflammatory cells and platelets to overcome circulatory shear forces and bind to the microvasculature [6-10].

Bone marrow derived haematopoetic and mesenchymal progenitor cells migrate to sites of injury in response to tissue damage [11-13], as do a host of circulating inflammatory cells such as neutrophils and monocytes [3,6]. Both bone marrow derived progenitor cells and inflammatory cells release reactive oxygen intermediates and proteases critical to matrix degradation and tissue invasion by these cells [6,14], and bone marrow derived progenitor cells can synthesize the collagens critical to early wound matrix formation [15]. In response to tissue injury inflammatory cells such as neutrophils and macrophages secrete a wide variety of cytokines such as IL-1 and TNF-a, whose pleiotropic effects include endothelial activation and a procoagulant state marked by the increased expression of endothelial adhesion molecules, the upregulation of leukocyte cytokine expression, and the proliferation of fibroblasts [3-6].

The growth factors released during this initial phase of inflammation trigger a cascade of additional growth factor release, initiating the second or proliferative phase starting 3 days after wounding and lasting for approximately 10 days. This phase is characterized by the further proliferation of migratory epithelial cells, endothelial cells, and fibroblasts, and the continued synthesis of a collagen rich matrix [3-6]. The last phase is the remodeling phase, a period of time in which the wound matrix matures and strengthens even further, and this phase occurs from days 8–12 onwards [3]. Note that many of the most important and critical aspects of the wound healing process are completed within one to two weeks after tissue injury.

Intriguing parallels between hematogenous cancer cell metastasis and the normal wound healing process have been recently discovered. After injecting mice with Lewis lung carcinoma cells or B 16 melanoma cells, Kaplan et al [16] found that tumor derived growth factors were able to choreograph the formation of specific cellular cluster sites or "premetastatic niches" where tumor cells could successfully metastasize to and develop into viable tumors. This premetastatic niche can be imagined as a scaffold upon which circulating cancer cells can adhere to and proliferate upon, or a nascent version of the mature tumor stroma. Bone marrow derived haematopoietic progenitor cells played a critical role in the formation of this niche, and their appearance at the sites of future metastases preceded the subsequent arrival of the circulating cancer cells and endothelial progenitor cells to these sites. The growth factors secreted by the circulating cancer cells triggered the rapid formation of these niches, and by day 14 after tumor cell implantation these cellular clusters could be visualized, with the subsequent development of viable micrometastases at these sites by day 23 [16].

The complex cellular behaviors involved in forming these pre-metastatic niches paralleled in some important respects the events that occur during the normal wound healing process. For example, bone marrow derived progenitor cells, known to migrate to sites of tissue injury during the wound healing process, left the bone marrow and helped to initiate the premetastatic niche in response to tumor derived factors [16]. Activated fibroblasts proliferated at these niches prior to the arrival of the bone marrow derived progenitors, and similar to their role during wound healing, synthesized the collagens and fibronectins critical to the binding and subsequent proliferation of circulating bone marrow derived progenitors [17] and inflammatory cells [18]. Furthermore, MMP 9 (matrix metalloprotein) expression was upregulated at these niches in concert with the appearance of the bone marrow derived progenitor cells [16], an upregulation noted to occur during the normal wound healing response and also facilitated by bone marrow derived progenitor cells [3,6,14]. Perhaps not surprisingly the time course in which some of these complex events occurred after tumor cell implantation was similar to the course of events that occur after tissue injury and during the initial inflammatory and proliferative phases of wound healing [3]. For example, fibronectin expression was increased at the sites of future metastasis by day 3 after tumor cell implantation; and resident stromal like fibroblasts proliferated at these niches in concert with the enhanced fibronectin expression by the first week after tumor inoculation [16].

The cancer derived signals activating this integrated response have not been clearly defined, but the precisely choreographed hijacking by circulating cancer cells of what appears in many respects to be a normal wound healing response to facilitate its own survival and metastatic spread is remarkable. This is perhaps not too surprising, as natural selection rarely reinvents the wheel, utilizing instead pre-existing biological patterns to solve new problems. Interestingly, the prompt administration of the monoclonal antibody bevacizumab, which targets vascular endothelial growth factor and is a potent inhibitor of the normal wound healing response, prevented the development of these premetastatic niches and cancer metastases in this study [16].

## Discussion

### Primary tumor removal activates the wound healing response and enhances the proliferation and survival of residual cancer cells

Surgical removal of the primary tumor and regional spread of the disease in the form of lymph node removal will trigger the first step of the wound healing process simply as a result of tissue injury. Ideally, any cancer remaining after a thorough surgical extirpation of clinically obvious disease will be within microspheres of metastatic deposits or the circulation. Circulating tumor cells must overcome shear forces imposed by the flow of blood and adhere to the microvasculature of a target tissue to survive and proliferate [19]. Immediately after surgery this process, relatively inefficient under normal circumstances [20], should be enhanced given an increase in vascular permeability and the enhanced expression of cell surface adhesive molecules such as the integrins and the selectins associated with the acute inflammatory response [6-10].

Activation of platelets and the coagulation pathway in response to tissue injury may also facilitate residual cancer cell metastasis, in particular, hematogenous metastasis. The binding of activated platelets to tumor cells has been shown to hinder natural killer cell mediated elimination of circulating tumor cells [21], and the destruction of circulating platelets with antiplatelet antibodies significantly reduces metastatic potential in several murine tumor models [22]. Activated platelets can significantly enhance the ability of circulating cancer cells to bind to the microvasculature [23], and platelet activation can trigger the release of platelet derived growth factors that stimulate cancer cell growth, angiogenesis, and the increased expression of cell surface adhesive molecules that can facilitate the microvascular arrest of circulating cancer cells [24,25].

Cancer cells not only can bind to activated platelets, they can also bind to a variety of coagulation factors that are released in large amounts during the initial inflammatory phase of wound healing, including fibrinogen, fibrin, tissue factor, and thrombin [26]. Cancer cell binding to coagulation factors and activated platelets can create a tumor cell embolus, limiting immune mediated recognition of the cancer cell [21] and facilitating cancer cell arrest in capillary beds and subsequent tissue extravasation [27]. Cancer cells not only can bind to the coagulation factors released in large amounts during the acute inflammatory phase of wound healing, they can synthesize many of these procoagulants themselves and activate the coagulation cascade [28], stimulating angiogenesis [29] and the formation of tumor stroma [30].

That the triggering of the coagulation cascade and platelet activation can facilitate cancer cell metastasis is not simply a theoretical consideration. Inhibition of tissue factor and thrombin significantly reduces hematogenous metastases in animal models [31]; and fibrinogen, released in large amounts during the initial phase of wound healing as result of the activation of the coagulation cascade, has been shown to be critically important to the ability of circulating cancer cells to metastasize, perhaps by enhancing their ability to adhere to the microvasculature [32]. What is perhaps even more disturbing is that the binding of activated platelets to the endothelium has been shown to target the recruitment of bone marrow derived progenitor cells through the release of the chemokine stromal derived factor or SDF-1 [33]. This potent molecule is not only chemotactic for the bone marrow derived haematopoietic progenitor cells shown to be critical to the development of the premetastatic niche [16], but also for cancer cells that often express the cognate receptor for SDF-1, CXCR4 [34].

The inflammatory and coagulation pathways activated by tissue injury will also, much like a domino effect, trigger the release of the growth factors characteristic of the proliferative phase of wound healing, including epidermal growth factor (EGF), fibroblast growth factor (FGF), platelet derived growth factor (PDGF), vascular endothelial growth factor (VEGF), and hepatocyte growth factor (HGF) [3-5]. These growth factors can augment the proliferation of residual cancer cells, fibroblasts, and endothelial cells, and some, such as HGF, facilitate the anchorage independent survival of circulating cancer cells [35,36] and are chemotactic for bone marrow derived progenitor cells [11]. Proliferating fibroblasts trigger the synthesis of the collagens that form the tumor stroma [3], and proliferating endothelial cells and activated platelets stimulate angiogenesis [3,6] and the binding of circulating inflammatory cells to the microvasculature through the upregulation of selectin expression [37].

The surgical removal of the primary tumor will not only trigger the wound healing response and an environment conducive to the metastatic spread of any residual cancer cells, it may well promote their hematogenous dissemination. Surgical manipulation of the primary tumor is associated with the shedding of tumor cells into the circulation in animal studies and an increase in metastatic proliferation [38,39]. In human beings, surgical removal of the primary tumor has been found to be associated with the seeding of cancer cells into the circulation in a number of different cancers, including lung [40,41], colorectal [42], breast [43,44], esophageal [45], prostate [46], gastric [47], and pancreatic cancer [48]. The presence of circulating cancer cells intraoperatively and during the perioperative period has been shown to be adversely correlated with both risk for relapse [49] and survival [40-42,50].

The presence of circulating cancer cells prior to surgical removal of the primary tumor has also been observed [51,52], but for the reasons discussed above the metastatic potential of circulating cancer cells is probably enhanced significantly after surgery given their enhanced ability to bind to the microvasculature as a result of the inflammatory events triggered by tissue injury as well as the often observed increase in their numbers during the perioperative period. And as described above in Kaplan's work [16], growth factors derived from circulating cancer cells were able to activate the molecular pathways critical to the formation of the "premetastatic niche."

Although it is clear that surgical wounding will activate the wound healing process and the release of inflammatory mediators and procoagulants that should theoretically stimulate the proliferation, survival and metastatic potential of residual cancer cells, there is evidence that primary tumor removal can also stimulate the proliferation of residual cancer cells via pathways independent of those triggered by surgical wounding alone. Important work done by Fisher and his colleagues over twenty years ago demonstrated that after the removal of a variety of different primary tumors in animals a growth factor was predictably upregulated in serum, causing residual cancer cells to convert from a non-cycling to a cycling phase, resulting in significant reductions in tumor doubling times and increased growth rates of distant metastases [53]. The cancer cells responding to this growth factor were thought to be a subpopulation of the total number of cancer cells remaining, as multiple injections of postoperative serum did not add to the observed increase in proliferation [54]. The increase in the proliferation of residual cancer cells was initially detected 24 hours after removal of the primary tumor, peaking at approximately 48–72 hours and lasting for several days [53,54]. The significant augmentation in proliferation noted by Fisher did not occur unless the primary tumor was removed, as sham surgery (amputation of a non-tumor bearing limb) failed to result in a significant increase in the proliferation of residual tumor cells [54].

How is this response triggered by primary tumor removal? There are multiple theories about this, and they need not be mutually exclusive. Primary tumor removal may change the predominant growth factor expression pattern in the host in such a way as to favor residual cancer cell proliferation. For example, Li and colleagues suggest that the increase in the proliferation of residual cancer cells after primary tumor removal results from reduced concentrations of circulating angiostatin [55]. Others have suggested that the immunosuppressive effects induced by surgical trauma could allow residual cancer cell proliferation [56]. But as discussed above in Fisher's work, sham surgery by itself did not result in a significant augmentation in the growth of residual cancer cells – the primary tumor had to be removed.

Another mechanism could be conceptualized by merging the findings of Kaplan [16] with the empirical observation that surgical removal of the primary tumor is often associated with the seeding of cancer cells into the blood. Such an event should theoretically set in motion the pathway uncovered by Kaplan's work, the pathway that it is being argued here is simply a triggering of the normal wound healing response. (Does this response require a critical mass of circulating tumor cells to be activated? And if so, what is that number?) Regardless of etiology, the important findings of Fisher showing that primary tumor removal is invariably associated with an increase in the proliferation of residual cancer cells suggests that this response is an adaptive mechanism utilized by residual cancer cells to survive, and it may also be a response that can be most effectively targeted when it is activated – the perioperative period

### Clinical trials evaluating perioperative chemotherapy

The empirical observations of Fisher, as well as some mathematical arguments discussed below [57], prompted clinical trials evaluating the effectiveness of administering chemotherapy perioperatively. These trials have been limited and have yielded conflicting results. In a randomized prospective trial in patients with breast cancer, Nissen-Meyer and colleagues compared the effects of perioperative cyclophosphamide with the same treatment administered three weeks after surgery. They found that both recurrence and mortality were significantly lower in the patients receiving the perioperative treatment [58]. Similarly, Sertoli and colleagues found that a single perioperative cycle of cyclophosphamide, fluorouracil, and epidoxorubicin yielded a statistically significant difference in relapse free survival in ER- patients, with no effect in hormonally positive patients [59]. An important follow-up study confirmed the initial results noted by Nissen-Meyer [60]. But in contrast to these findings, a large trial in patients with early stage breast cancer did not find a statistically significant improvement in survival utilizing cyclophosphamide perioperatively [61].

What are we to make of these disparate findings? Given the very limited trials to date it remains an unanswered question as to whether perioperative chemotherapy treatment is more effective than chemotherapy started only after the surgical wound has healed. It is important to note that adjuvant chemotherapy of any kind has only been confirmed to be effective over the last fifteen years [62-64], and historically there has been real doubt whether chemotherapy was of any benefit in the adjuvant setting. But despite the fairly recent and very real success of chemotherapy in improving cure rates in the adjuvant setting, its ineffectiveness in the cure of metastatic cancer sustains the pervasive nihilism that persists towards the disease, perhaps limiting our ability to see what is possible with the increasingly effective treatments now available to us.

### Theoretical rationale for targeting residual cancer perioperatively

Conceptually there are two ways of thinking why adjuvant treatments should be more effective when administered during the perioperative period rather than waiting 30 days or more after surgery. One is based on the kinetics of cancer cell growth; the other on what I would describe as the kinetics of the formation of the cellular infrastructure necessary to support residual cancer cell survival and proliferation. I shall take each of these concepts in turn.

The theoretical rationale for the increased effectiveness of treating cancer perioperatively on the basis of cancer kinetics is derived from the empirical observations of Fisher and a few fundamental mathematical arguments. Goldie and Coldman argued that delays in starting therapy after surgical resection of the primary tumor result in an increase in resistant tumor cells, and that the lowest number of resistant tumor cells should be present immediately after surgical debulking of the cancer and during the perioperative period [57]. Gompertzian growth curves applied to the kinetics of tumor growth predict that microfoci of cancer deposits (the situation presumed to exist after surgical removal of the primary tumor) will grow rapidly owing to a higher percentage of cells in the growth phase [65]. Given that rapidly cycling tumors are often very sensitive to chemotherapy agents [66], and the rare metastatic solid cancers curable with chemotherapy have high rates of proliferation and sensitivity to chemotherapeutic agents [67], there are indeed good arguments based strictly on kinetics for why the perioperative administration of chemotherapy should prove more effective. Moreover, it is intuitive that for any finite log kill ratio one can achieve with therapy, tumor eradication or cure is more likely when the number of cells that have to be eradicated is less than the log kill ratio of the therapy used. (The longer one waits before initiating therapy, given the survival of residual cancer cells that are proliferating, the greater is the number of cells that have to be eradicated to effect cure.)

The importance of starting therapy quickly to avoid being overwhelmed by a pathogenic multiplicative process is already known in infectious disease management. For example, one of the most important determinants of survival in sepsis is the prompt administration of effective antibiotics [68]. Similarly, early administration of effective antiviral therapy to macaques after exposure to SIV (a primate model of HIV) has been shown to reduce significantly the risk of acquiring subsequent infection, while delays in initiating therapy are correspondingly less effective [69]. This basic science information has been translated into the treatment of individuals exposed to HIV. Prompt treatment with effective antivirals after percutaneous exposure to HIV significantly reduces the risk of subsequent infection [70]. Although the growth kinetics of a rapidly replicating virus such as HIV are more rapid than even the most aggressive of cancers, and the disease processes are different with different challenges, conceptually they present a similar problem: Both are associated with the inexorable increase of the cancer cell or pathogen over time, with a correspondingly increased difficulty in eradication the later effective therapy is initiated. In fact, treatment timing may be even more important in the case of cancer given our inability to cure most solid cancers once they have spread much beyond the primary tumor.

Despite the sound theoretical rationale for the perioperative treatment of cancer on the basis of the kinetics of residual cancer cell growth, it may be by targeting the wound healing pathway and the formation of the cellular infrastructure essential to residual cancer cell survival and proliferation where perioperative treatment might prove most beneficial. Based upon the wound healing pathway described above the following events are occurring during the 30 days or more that we now wait before starting adjuvant therapy: angiogenesis; residual tumor cell and fibroblast cell proliferation; collagen, thrombin, and fibrinogen deposition and matrix formation; upregulation of cell surface molecules such as the integrins and the selectins enhancing circulating cancer cell adherence to the microvasculature; and bone marrow derived progenitor and endothelial cell migration to potential future metastatic sites and the formation there of "premetastatic niches."

The biological requirements integral to the survival of a cancer cell are likely very different when it is embedded within a mature tumor microenvironment – composed of stromal fibroblasts, myofibroblasts, and mature matrix – than when it is pushed into the circulation or within immature micrometastatic deposits. Cancer cells within a mature tumor microenvironment are more likely to be resistant to our chemotherapies for multiple reasons, both conceptual and empirical. One reason is based strictly on biophysical constraints imposed by volume and surface area, as cancer therapies are not likely to reach cancer cells within dense primary tumor stroma as easily as they would if those cells were within nascent tumor microenvironments [71]. And once a cancer cell is safely embedded within the tumor stroma it becomes significantly more resistant to medical treatments. For example, cancer cell adherence to the matrix substrata composed of collagen and fibronectin is associated with cancer cell survival and resistance to drug therapy in multiple cancers [72,73].

Disrupting the wound healing process during the perioperative period may also improve our ability to eradicate residual cancer stem cells. Recent discoveries suggest that many tumors are composed of a heterogenous population of cancer cells with a subpopulation of cancer stem cells critical to the survival of the tumor and more resistant to chemotherapy [74]. (Are the residual cancer cells noted by Fisher to proliferate after primary tumor removal more likely to be cancer stem cells?). If we can disrupt the wound healing process and the production of the cellular architecture necessary for residual cancer cell survival and proliferation during the perioperative period, it should theoretically enhance our ability to eradicate residual cancer stem cells, regardless of their sensitivity to chemotherapy.

### Back to the future: Targeting cancer perioperatively

The limited trials to date testing the effectiveness of treating cancer perioperatively have been evaluated almost exclusively utilizing chemotherapeutic agents alone. But not only do we have a better selection of chemotherapeutic agents today than when the perioperative trials discussed above were conducted, we also have highly effective targeted agents that are capable of disrupting various components of the wound-healing pathway, including angiogenesis [75]. These therapies have already proven effective in treating metastatic cancer and prolonging survival [76]. But their true value is likely to be found in the adjuvant setting, and in particular, during the perioperative period and the time when wound healing pathways can be effectively disrupted before residual cancer cells are able to establish new micrometastatic deposits.

The critical importance of platelet activation and the triggering of the coagulation pathway in the pathophysiology of cardiovascular disease has spurred research that has developed effective drugs interfering with various stages of this process. But more and more experimental evidence suggests that platelet activation and the triggering of the coagulation cascade may also be very important in the pathophysiology of cancer, particularly, in facilitating the hematogenous spread of cancer. Given the abundance of medicines that target platelets and vaious components of the coagulation cascade now available, we can design rational clinical trials that could utilize some of these agents immediately after the primary tumor has been removed. For example, the use of heparin, both unfractionated and low molecular weight heparin, has been shown to hinder the binding of activated platelets to tumor cells [77]. Something as simple as starting cancer patients on anticoagulation with heparin after the primary tumor has been removed may improve cure rates in a wide variety of solid malignancies. And there is increasing experimental evidence that by targeting certain integrin receptors important in platelet activation an effective disruption of tumor cell-platelet interactions can be achieved [78].

Just as combination chemotherapy in general has proven more effective in treating cancer than single agents, it might be wise to employ multiple agents targeting various components of the wound healing and inflammatory pathway to target cancer perioperatively. Therapeutic agents are already in development that target acute inflammatory mediators such as TNF-a or IL-1 [79], and their use immediately after surgery might be able to disrupt the subsequent growth factor release essential to residual cancer cell survival and proliferation. The SDF-1/CXCR-4 axis facilitates the directed migration not only of cancer cells but also the bone marrow derived progenitors implicated in the development of the premetastatic niche [80]. This critical communication pathway could also be targeted, perhaps most effectively during the perioperative period and before residual circulating cancer cells and cancer stem cells can establish new micrometastatic deposits.

I have said nothing about how treating cancer by targeting wound healing pathways perioperatively might impede surgical wound healing. Although this issue is non-trivial, it is difficult to imagine that it should be an obstacle impossible to overcome with aggressive monitoring and using effective artificial wound closure techniques. One could test the safety of targeting the systemic wound healing response by administering one or more of the agents discussed above perioperatively in animals subjected to surgical procedures mimicking the morbidity associated with primary tumor removal. Although surgical wound healing might well be delayed, and some complications result, I suspect that most animals would survive these treatments and return to normal health after being administered some of the compounds discussed above.

It is critically important to remember that persons who undergo surgery to remove a cancerous tumor are at grave risk of dying from cancer, and we must not allow the current clinical focus on wound healing to blind us to this vital consideration. This is particularly true since residual cancer cells may be utilizing the wound healing pathway to facilitate their survival and metastasis, and clinically documented metastatic cancer is almost always a death sentence. It may be during the perioperative period and well before the surgical wound heals where our best opportunity to eradicate any residual cancer lies. Clinical trials are urgently needed to test the safety and efficacy of perioperative cancer treatments that not only target the cancer cell but the wound healing pathway utilized by the cancer cell to proliferate and metastasize.

## Summary

1. New discoveries have revealed important parallels between wound healing and metastasis, and cancer cells may rely on these pathways to survive and metastasize.

2. Primary tumor removal activates wound healing pathways as a result of the surgical trauma, the removal of the primary tumor, and the seeding of cancer cells into the circulation.

3. These pathways are activated immediately after surgery, with the peak increase in the proliferation of residual cancer cells occurring within 24–72 hours after primary tumor removal.

4. Allowing wound healing to occur before initiating therapy may be facilitating metastatic spread of the cancer and compromising the subsequent effectiveness of that therapy.

5. Clinical trials are needed to test the safety and efficacy of administering adjuvant therapies perioperatively that target both residual cancer cells as well as the wound healing pathways utilized by the cancer to metastasize.

## Competing interests

The author declares that they have no competing interests.

## Pre-publication history

The pre-publication history for this paper can be accessed here:

http://www.biomedcentral.com/1471-2407/9/118/prepub
